# Confirmatory factor analytical study of the WHOQOL-Bref: experience with Sudanese general population and psychiatric samples

**DOI:** 10.1186/1471-2288-7-37

**Published:** 2007-08-01

**Authors:** Jude U Ohaeri, Abdel W Awadalla, Abdul-Hamid M El-Abassi, Anila Jacob

**Affiliations:** 1 Department of Psychiatry, Psychological Medicine Hospital, Gamal Abdul Naser Road, P.O. Box 4081, Safat, 13041 Kuwait; 2 Department of Psychiatry, Faculty of Medicine, Kuwait University, Kuwait; 3 Statistics Unit, Manpower Planning General Republic Program, Box 2822, Safat, 13029 Kuwait

## Abstract

**Background:**

The widespread international use of the 26-item WHO Quality of Life Instrument (WHOQOL-Bref) necessitates the assessment of its factor structure across cultures. For, alternative factor models may provide a better explanation of the data than the WHO 4- and 6-domain models. The objectives of the study were: to assess the factor structure of the WHOQOL-Bref in a Sudanese general population sample; and use confirmatory factor analysis (CFA) and path analysis (PA) to see how well the model thus generated fits into the WHOQOL-Bref data of Sudanese psychiatric patients and their family caregivers.

**Method:**

In exploratory factor analysis (FA) with all items, data from 623 general population subjects were used to generate a 5-domain model. In CFA and PA, the model was tested on the data of 300 psychiatric outpatients and their caregivers, using four goodness of fit (GOF) criteria in Analysis of Moment Structures (AMOS). In the path relationships for our model, the dependent variable was the item on overall QOL (OQOL). For the WHO 6-domain model, the general facet on health and QOL was the dependent variable.

**Results:**

Two of the five factors ("personal relations" and "environment") from our FA were similar to the WHO's. In CFA, the four GOF criteria were met by our 5-domain model and WHO's 4-domain model on the psychiatric data. In PA, these two models met the GOF criteria on the general population data. The direct predictors of OQOL were our factors: "life satisfaction" and "sense of enjoyment". For the general facet, predictors were WHO domains: "environment", "physical health" and "independence'.

**Conclusion:**

The findings support the credentials of WHO's 4-domain model as a universal QOL construct; and the impression that analysis of WHOQOL-Bref could benefit from including all the items in FA and using OQOL as a dependent variable. The clinical significance is that by more of such studies, a combination of domains from the WHO models and the local models would be generated and used to develop rigorous definitions of QOL, from which primary targets for subjective QOL interventions could be delineated that would have cross-cultural relevance.

## Background

The WHO developed a 100-item quality of life (QOL) assessment instrument, the WHOQOL-100 [1], based on the definition of subjective QOL as individuals' perception of life in the context of the culture and value system in which they live and in relation to their goals, expectations, standards and concerns. A 26-item version, the WHOQOL-Bref, was derived from there [2]. This instrument deals with subjective QOL as distinct from objective QOL [3]. This is in line with the trend in the literature, whereby in the assessment of QOL, more attention has been focused on an individual's subjective feelings on aspects of life, rather than the traditional views of success and assessments of material well being [4]. The items ask about satisfaction with circumstances of living in areas such as the presence of physical pain, need for medical treatment for daily functioning, enjoyment of life, money for needs, personal relations, transport, etc. There are five Likert-type response options, ranging from "very dissatisfied" (score of 1) to " very satisfied" (score of 5), with higher scores denoting higher QOL. The instrument was developed in a wide range of languages in different cultural settings and yields comparable scores across cultures [2]. It is made up of domains (or dimensions) and facets (or sub domains). Domains are broad groupings (e.g., physical, psychological health) of related facets. The items on "overall rating of QOL" (OQOL) and subjective satisfaction with health, are not included in the WHO domains, but are used to constitute the general facet on health and QOL. There are two models of the WHOQOL-Bref. The initial model was fashioned in line with the WHOQOL – 100[1] to have six domains, namely, physical health, psychological health, level of independence, social relationships, environment, and spiritual. To derive the second (4 – domain) model, the domain of level of independence was merged with that of physical health, while the "spiritual" was merged with the psychological.

The widespread international use of the WHOQOL-Bref provides a compelling rationale to assess its factor structure across culturally diverse groups. Although there are many reports of the 4- and 6-domain models [2,4], these studies did not investigate the possibility that alternative factor models may provide a better explanation of the data. Hence, in a Nigerian study in which all the 26 items were entered into factor analysis, the resulting eight factors were found to have better structural integrity indices than the WHO's models in confirmatory factor analysis (CFA), and provided a more succinct definition of QOL than could be derived from the WHO factors [5]. This possibility, that using all the items of the WHOQOL-Bref in factor analysis could lead to the generation of factors from local data sets that are of comparable usefulness to the established WHO domains, requires further exploration. In this way, we could compare QOL dimensions across cultures (i.e., using the WHO domains), while providing additional information about local QOL characteristics (by using factors generated from local data sets). For instance, in a Korean path analytic (PA) study, it was found that the physical and psychological domains made more significant contributions to explaining the variance in QOL, while the independence and spiritual domains made less impact. The authors interpreted this to imply that Koreans regard independence, individualism and spirituality, the weighted values in Western societies, to be less important [4].

An additional value of factor analytic studies is that, we could gain more insight into the factor structure of the instrument across cultures, and thereby generate factors that could be used to articulate more rigorous definitions of QOL. From these definitions, targets for subjective QOL interventions could be delineated that would have cross-cultural relevance.

Based on the above premises, we collected data, using the WHOQOL-Bref, from three segments of the Sudanese population. These were: a general population sample, community living persons with psychiatric disorders in stable condition, and the family caregivers of the patients.

The objectives of our study were:

- to assess the factor structure of the WHOQOL-Bref in a Sudanese general population sample;

- to use confirmatory factor analysis (CFA) and see how well the factors from the general population fit into the WHOQOL-Bref data of Sudanese psychiatric patients and their family caregivers;

- to compare the WHO models with the Sudanese general population model, using CFA;

- to use path analysis (PA) to compare the structural integrity of the domain relationships generated by the WHO models, with that generated by the model from the Sudanese general population;

- to assess the factors that predict the rating of the item on overall quality of life (OQOL).

In other words, as recommended by the structural equation modeling (SEM) technique [6], our model was developed using the general population data as the calibration data sample, and then confirmed using the data from the psychiatric patients and their family caregivers as the independent validation samples.

The following research questions were explored: (1) Does exploratory factor analysis (FA) of the Sudanese general population data generate factors/dimensions that are similar to those of the WHO 4- and 6-domain models; (2) Does the model generated from the Sudanese general population provide a superior fit to the data from the psychiatric patients and their family caregivers, than the WHO models?

Based on previous experience [5], we hypothesized that the Sudanese general population data would yield different factors from the WHO's, and that the model constituted by these factors would have a better fit to the Sudanese data, than the WHO models.

## Method

The procedure for data collection, the clinical and socio-demographic characteristics, and the QOL characteristics of the patients, caregivers and control group have been described in detail elsewhere [7,8]. The patients were consecutive attendees at the psychiatric clinics of various hospitals in Sudan, with stable and unequivocal case note psychiatric diagnoses based on the WHO ICD-10, the official classification system of the country (equivalent to the corresponding DSM-IV diagnoses). The patients were accompanied by family members who could independently complete the questionnaires in Arabic. Of the 300 patients, 99 had schizophrenia, 120 had major affective disorders, and 81 had non-psychotic mild to moderately severe mental disorders (anxiety disorders, and mild-moderately severe depression). The mean age of the patients was 33.8 (10.3) years, 194 (64.6%) had at least high school education, 82 (27.3%) were married, and 92 (44.0%) were formally employed. The 300 family caregivers (150 men, 150 women) were aged 42.7(12.9) years, 194 (64.7%) had at least high school education, 193 (64.3%) were married, and 127 (43.8%) were formally employed. Patients and caregivers each completed the WHOQOL-Bref privately, with trained research assistants nearby to assist them.

The study was carried out in compliance with the Helsinki Declaration. Ethical approval for the work was obtained from the University of Ribat-Criminology and Social Studies Research Institute, Khartoum, Sudan, and the Faculty of Medicine, Kuwait University. The authorities in each hospital approved the study. In addition, patients and their family caregivers gave verbal informed consent to participate in the study. They were duly informed that there would be no negative consequences for declining to participate. As is well known in our culture for such non-invasive studies, all families approached freely consented to participate in the study, especially as the approach was made by clinic staff in charge of the cases.

For the general population sample, we recruited subjects in living conditions similar to those of the patients. They also gave verbal informed consent to participate in the study. In order to fulfill the requirement for adequate sample size in structural equation modelling (SEM) [9], we recruited 623 general population subjects. In doing this, we sought to have a general population sample that would reflect the independently living, disease-free adult age group proportions in the Sudanese general population. The general population sample consisted of 623 subjects (46.5% men, 52.8% women; gender data missing for four subjects) who volunteered to complete the questionnaire. Their mean age was 26.1 (7.9) years, 567 (91.0%) had at least high school education, 103 (16.5%) were married, and 261 (41.9%) were formally employed. They were selected as a calibration sample for the SEM operations.

### Data analysis

Data were analyzed by the Statistical Package for Social Sciences (SPSS) version 11. Structural equation modelling (SEM) operations (confirmatory factor analysis – CFA and path analysis – PA), were done by Analysis of Moment Structures (AMOS) [6].

First, exploratory factor analysis (FA) was done with the general population data by principal component analysis, with Varimax rotation for factors with Eigen values above one. In the initial FA operation, all the 26 items of the WHOQOL-Bref were utilized (i.e., including "overall rating of QOL" – OQOL, and satisfaction with health). In the second FA operation, only 24 items were used (i.e., excluding OQOL and health satisfaction, as in the WHO's approach). However, the factors resulting from the later FA were not conceptually meaningful; and hence all subsequent analyses were based on the factors from the initial FA operation. Second, for each of the three populations (i.e., general population, psychiatric patients, and caregivers of psychiatric patients), QOL domain scores were generated by summing up the scores for items of the WHOQOL-Bref in each of the domains of the WHO models, as well as the 5 and 6 domains resulting from our FA operation [5]. Third, the internal consistency of each domain was assessed by Cronbach's alpha values, in which the acceptable level was at least 0.7, following standard guidelines. Cronbach's alpha values of the questionnaire for the responses of all subjects were high: 0.88, 0.93, and 0.92, respectively, for the general population, psychiatric patients and caregivers.

Fourth, CFA was then used to compare the "goodness of fit" (GOF) of the model resulting from our FA operation, with the WHO models for each of the three populations. Fifth, using a series of multiple regression analyses and Pearson's correlations (with OQOL as dependent variable and the factors from our general population FA as independent variables), we generated a model of relationships among the factors (using the general population data). Sixth, we tested the structural integrity of this model in PA, for each of the three populations[6]. In doing this, we analyzed separately, the model resulting from our original six factors (our 6-domain model) and that resulting from combining our fifth and sixth factors (our 5-domain model). A similar PA was done for the WHO models (using a path model generated from the general population data), but using the general facet on health and QOL as the dependent variable. Our "goodness of fit" estimation method was the generalized least squares method (GLS) [6].

### Goodness of fit criteria (GOF)

There are varying suggestions in the literature about the number, type and cut-off values for GOF required to be reported [10]. A popular recommendation is to present three or four indices from different areas. Accordingly, we report the following fit indices because of their popularity in the literature:

- Relative chi-square (X^2^/df), is the chi-square fit index divided by degrees of freedom, in an attempt to make it less dependent on sample size. (cut-off values for good fit: <2 to 5).

- Goodness-of-fit index (GFI) and adjusted GFI (AGFI) are chi-square based calculations independent of degrees of freedom (cut-off value ≥ 0.9)

- Root mean square error of approximation (RMSEA) is based on predicted versus observed co-variances but penalizing for lack of parsimony (or simplicity), in assessing a model's amount of error. It is popular because it does not require comparison with a null model (cut-off values: 0.05 to 0.08)

- Akaike Information Criteria (AIC), is based on information theory. It is used to compare non-hierarchical and hierarchical (nested) models. AIC close to zero reflects good fit; and between two AIC measures, the lower one reflects the model with the better fit.

In summary, we had four models that were all compared by CFA and PA in the three distinct populations (i.e., 623 general population subjects, 300 psychiatric patients, and caregivers of psychiatric patients). The models were as follows:

- Our six-domain model resulting from our FA operation on the data from 623 general population subjects;

- Our 5-domain model resulting from combining the fifth and sixth factors from the above FA operation;

- WHO's 4-domain model;

- WHO's 6-domain model.

## Results

Factor analysis (Tables 1 and 2): In exploratory factor analysis of the general population data, using all the items of the WHOQOL-Bref, six conceptually meaningful factors/domains emerged, accounting for 54.5% of the variance. Of these, the first four had at least three items each, and were thus stable. In order to enhance stability and conceptual meaning, the fifth factor (with two items) and sixth (with one item) were merged to produce a conceptually meaningful factor of "physical and mental health". It is noteworthy that parsimony (or simplicity) of the factors was observed, as each item of the questionnaire loaded on only one factor, with a minimum item loading of 0.45. In view of the constituent items of the remaining factors (see Table 1), they were labelled, successively (factors 1 to 4), "life satisfaction", "sense of enjoyment", "environment", and " social relations". It is to be noted that our "social relations" factor was defined by the same three items which constitute the WHO model of the same label. In addition, our "environment" domain appears to be a tighter definition of the WHO domain of same label (with five of the eight items that constitute the WHO domain) (Table 1). The internal consistency values of the domains are shown in Table 2. While an appreciable number of our domains (from our 5-domain model) and the WHO 4-domain model reached the 0.7 level, none of the three domains that distinguish the WHO 6-domain model reached the 0.7 level. In other words, the factors of our 5-domain model and WHO's 4-domain model had mostly acceptable internal consistency, while the factors of the WHO 6-domain model had rather low internal consistency in our general population data.

**Table 1:** Factor analysis using WHOQOL-Bref data from 623 Sudanese general population data

WHOQOL-Bref items	F1: Life satisfaction	F2: Sense of enjoyment	F3: Environment	F4: Social relations	F5: Physical & mental health
Energy for life	0.68				
Accept body appearance	0.65				
Able to concentrate	0.62				
Satisfied with information	0.59				
Safety in daily life	0.57				
Activities of daily living	0.57				
Feel life meaningful	0.55				
Satisfaction work capacity	0.54				
Satisfaction with self	0.53				
Able to get around	0.49				
Overall QOL		0.75			
Overall health satisfaction		0.67			
Sleep satisfaction		0.57			
Enjoyment of life		0.46			
Leisure opportunities		0.46			
Access to health services			0.73		
Transport satisfaction			0.73		
Money for needs			0.58		
Living place satisfaction			0.56		
Physical environment			0.49		
Support from friends				0.61	
Sexual satisfaction				0.57	
Personal relations				0.57	
Need for medical treatment					0.85
Freedom from pain					0.83
No negative feelings					0.67**

Note: ** Negative feelings loaded on Factor 6, but added now to Factor 5 for conceptual meaning and good fit in CFA

**Table 2:** Internal consistency (Cronbach's alpha), Eigen values and percent of variance for the factors

Factor labels	No of items	Cronbach's alpha	Eigen value	% of variance
A: factors from 623 general population data				
F1: Life satisfaction	10	0.85	7.11	27.33
F2: Sense of enjoyment	5	0.73	2.01	7.71
F3: Environmental health	5	0.68	1.45	5.58
F4: Social relations	3	0.45	1.38	5.29
F5: Physical and mental health**	3	0.52	1.18,1.06	4.54, 4.08
Total: for all items together	26	0.88		54.54
B: WHO 4-domain model on general population				
F1: Physical health	7	0.71		
F2: Psychological health	6	0.71		
F3: Social relations	3	0.45		
F4: Environment	8	0.74		
C: WHO 6-domain model on general population				
F1: Physical health	3	0.40		
F2: Psychological	5	0.61		
F3: Independence	4	0.58		

• *Items for WHO 6-domain environment and social relations are identical with 4-domain factors; F6 is "life meaningful"

• ** For factors from general population: original F5 (2 items, alpha value = 0.685, Eigen value, 1.18; % variance = 4.08), merged with F6 (Eigen value, 1.06; % variance = 4.08). **** Cronbach's coefficient's value is associated with the number of items in a scale.

### Confirmatory factor analysis (Table 3)

**Table 3:** Confirmatory factor analysis results: estimations by generalized least squares (GLS)*

Structural fit indices	Using 6F & 5F models from 623 general population subjects						Applying WHO 4- & 6-domain models					
	General popn subjects		Psychiatric patients		Family caregivers		Gen popn subjects		Psychiatric pts		Family caregivers	

	6F model	5F model	6F model	5F model	6F model	5F model	4d model	6d model	4d model	6d model	4d model	6d model

No of parameters	57	56	58	57	57	57	52	54	51	53	52	54
Discrepancy (X^2^)	936.6	925.8	638.4	623.4	688.0	717.4	859.3	947.6	548.9	556.4	611.9	664.4
DF	295	297	299	297	298	300	250	249	252	253	251	250
Discrepancy/DF	3.18	3.12	2.14	2.09	2.31	2.39	3.44	3.81	2.18	2.19	2.44	2.66
GFI	0.88	0.89	0.84	0.84	0.82	0.82	0.89	0.87	0.85	0.84	0.83	0.81
Adjusted GFI	0.86	0.86	0.81	0.81	0.79	0.78	0.86	0.85	0.82	0.82	0.79	0.78
AIC	1050.6	1037.8	754.4	737.4	802.0	831.4	963.3	1053	650.9	662.4	715.9	772.4
RMSEA	0.059	0.058	0.062	0.061	0.066	0.068	0.063	0.067	0.063	0.063	0.069	0.075

NOTES: DF = degrees of freedom; 6F mod = 6-factor model; 5F mod = 5-factor model; 4d mod = 4-domain model; 6d mod = 6-domain model; GFI = goodness of fit index; FI = fit index; RMSEA = root mean square error; AIC = Akaike index; Gen popn = general population

* Ideal fit indices are: Discrepancy/Df < 5; GFI, AGFI ≥ 0.9; RMSEA = 0.05 – 0.08; lower AIC

All the models had RMSEA values less than 0.08 (Table 3), an indication that they did not have significant error. All the models performed well for the fit index, relative chi-square (X^2^/df), with values that ranged from 2.09 (data from application of our general population 5-domain model on psychiatric patients) to 3.81 (data from application of WHO 6-domain model on the general population sample), well below the recommended cut-off value of 5. Although the GFI values were less than the required 0.9, they were appreciably high, being above 0.8. The AGFI values were mostly similar, with the least being 0.78. Using the AIC values, the best performing models were: application of the WHO 4-domain model on the psychiatric patients (650.9), application of the WHO 4-domain model on the family caregivers (715.9), and application of our 5-domain model on the psychiatric patients (737.4). Combining the four criteria for goodness of fit earlier highlighted, the two most fitting models were, our five-domain model on the psychiatric patients, and WHO's 4-domain model on the psychiatric patients.

### Path analysis (Table 4 and Figs 1 &2)

**Figure 1:**
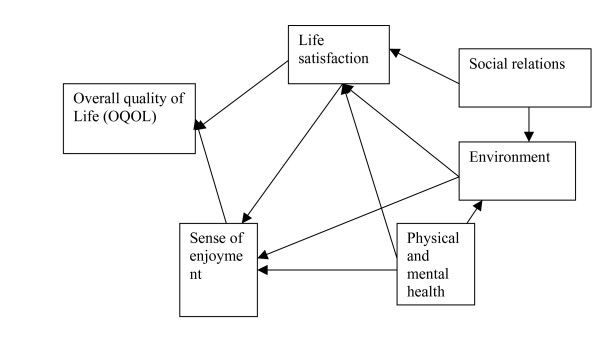
**Path relationships within the 5 domains from Sudan general population data**. Primary predictors of OQOL: "life satisfaction", standardized beta = 0.82; "sense of enjoyment", standardized beta = 0.74."Social relations", "environment", "physical/mental health", impacted on OQOL indirectly through "life satisfaction" and "sense of enjoyment", as shown in the path diagram.

**Figure 2:**
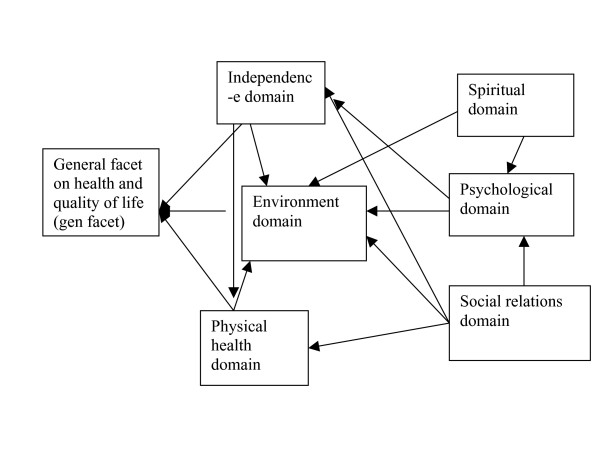
**Path relationships within the WHO 6-domain model derived from Sudanese general population data**. The primary (direct) predictors of "gen facet" were: "environment", standardized beta = 0.18; "physical health", beta = 0.26; and "independence, beta = 0.18". The " psychological", " social relations" and "spiritual" domains impacted on "gen facet" indirectly through effects on the primary predictors, as shown in the path diagram.

**Table 4:** Path analysis results: estimations by generalized least squares (GLS)*

Structural fit indices	Using 6F & 5F models from 623 general population subjects						Applying WHO 4- & 6-domain models on					
	General population subjects		Psychiatric patients		Family caregivers		Gen population Subjects		Psychiatric pts		Family caregivers	

	6F model	5F model	6F model	5F model	6F model	5F model	4d model	6d model	4d model	6d model	4d model	6d model

No of parameters	16	15	15	16	16	15	12	21	12	21	12	21
Discrepancy (X^2^)	62.42	9.26	82.69	63.19	63.24	46.08	11.82	121.51	32.12	95.72	36.27	96.28
DF	12	6	12	6	12	6	3	7	3	7	3	7
Discrepancy/DF	5.2	1.54	6.89	8.7	5.27	7.68	3.94	17.36	10.71	13.67	12.09	13.76
GFI	0.97	0.99	0.88	0.94	0.94	0.95	0.99	0.94	0.96	0.91	0.95	0.91
Adjusted GFI	0.93	0.98	0.72	0.79	0.86	0.82	0.96	0.78	0.78	0.63	0.76	0.63
AIC	94.4	39.3	114.7	82.5	95.2	76.1	35.8	163.5	56.1	137.7	60.3	138.3
RMSEA	0.08	0.03	0.14	0.16	0.12	0.15	0.07	0.16	0.18	0.21	0.22	0.21

NOTES: DF = degrees of freedom; 6F mod = 6-factor model; 5F mod = 5-factor model; 4d mod = 4-domain model; 6d mod = 6-domain model; GFI = goodness of fit index; RMSEA = root mean square error; AIC = Akaike index; Gen popn = general population

* Ideal fit indices are: Discrepancy/Df ≤ 5; GFI, Adjusted GFI ≥ 0.9; RMSEA = 0.05 – 0.08; lower AIC

As earlier indicated, the path models were generated using the general population data. In step-wise regression analysis, we used the factors derived from our general population data as the independent (predictor) variables, and the item of overall quality of life (OQOL) as the dependent variable. The only direct predictors of OOQL were "life satisfaction" (factor 1) (standardized Beta = 0.82, and " sense of enjoyment" (factor 2) (Beta = 0.74). The remaining factors made their contributions to OQOL through their impact on these two factors (Fig 1). For the WHO 6-domain model, the direct predictors of the general facet on health and QOL were, "environment" (Beta = 0.32), "physical health" (Beta = 0.26) and " independence" (Beta = 0.18), with the remaining factors making their input on QOL through their impact on the three factors (Fig 2). Surprisingly for this conservative culture, the so called " spiritual" factor (constituted by the item on life being meaningful) was not a direct predictor of QOL. For the WHO 4-domain model, the direct predictors of QOL were " physical health" (Beta = 0.39) and " environment" (Beta = 0.32).

Unlike in the CFA data, most path model relationships (except our 5-domain model on the general population, 0.03; and the WHO 4-domain model on the general population, 0.07) had RMSEA values over 0.08, indicating significant levels of error. In addition, the X^2^/df value was below five for only our 5-domain model applied on the general population, and the WHO 4-domain model applied on the general population (3.9) (Table 4). However, the GFI values were impressive, with virtually all of them over the required 0.9 threshold. The AGFI values reached 0.9 level for three path models applied on the general population data, namely, the WHO 4-domain model, and our two models. Using the AIC index, the three most plausible models were, the WHO 4-domain model on the general population(35.8), our 5-domain model on the general population (39.3), and the WHO 4-domain model on the psychiatric patients (56.1).

In summary, combining the four goodness of fit (GOF) criteria, only two path models met all the criteria for "good fit", namely, our 5-domain model on the general population, and the WHO 4-domain model on the general population data.

## Discussion

Although our study shared the same limitations as similar studies [1,2,4] in the sense that the patients were not representative of the Sudanese general population of psychiatric patients, our data fulfilled the conditions for structural equation modelling, by the fact that our sample sizes were adequate with respect to a 26-item questionnaire[9]. While the path analytical method using the summary scores (i.e., manifest variables) of each of the domains is appropriate for path analysis, an alternative method would have been to treat each of these as a latent variable, with the individual items as the measured variables. This has the potential to disattenuate the measurement error associated with each domain, and would probably yield a better estimate of the relationships among them. In this regard, we suggest that the inclusion of RMSEA as a fit index has helped to strengthen the rigor of our methodology.

As recommended by the SEM technique, we generated our models from an appropriate calibration data sample, and tested them in two independent validation data samples. Another strength of our study is that we compared locally generated models with the WHO models. The robustness of our findings is shown by the fact that they were based on four goodness of fit (GOF) criteria and applied on three different population groups. But our findings should be interpreted in the light of the knowledge that path analysis (PA) cannot be used to establish causality or even to determine whether a specific model is correct; it can only determine whether the data are consistent with the model [6,9]. In addition, our objective was not to validate the WHOQOL-Bref as an instrument of measurement.

With respect to our research questions and hypothesis, the highlights of our findings are first, that exploratory factor analysis of our general population data generated a domain structure that included two factors ("personal relations" and "environment") which are similar to those of the WHO's. Second, our 5-domain model and WHO's 4-domain model had similar fit indices in CFA in the three population groups. Third, in PA, the validity or structural integrity of these domains in the general population data was proven by the fact that the relationships within these domains adequately fulfilled the four GOF criteria. These findings indicate the cross-cultural salience of the dimensions of "personal relations" and "environment" in the definition of subjective QOL, and add robustness to the credentials of WHO's 4-domain model as a universal construct of subjective QOL. The finding about the structural integrity of our 5-domain model indicates that it is valid to analyze the WHOQOL-Bref by factor analysis using all the items, and that the item on satisfaction with overall quality if life (OQOL) can also be used as a dependent variable.

The theoretical support for our recommendation of OQOL as a dependent variable is as follows. In a critical appraisal of QOL instruments, Gill and Feinstein [11] highlighted the need for two global ratings, one on OQOL and the other on health – related QOL. They recommended that the item on OQOL be analyzed separately, instead of being combined with that on health – related QOL (as in the WHOQOL-Bref). In advancing this position, they noted that, OQOL may encompass not only health – related factors, but also many non – medical phenomena, such as employment, family relationships and spirituality.

The high "goodness of fit" performance of our 5-domain model and WHO's 4-domain model in the general population data, implies that the predictors of QOL that we derived from these models in multiple regression analysis are worthy of note. From our analysis of the WHO 4-domain model, the direct predictors of QOL were " physical health" and " environment", while " psychological health" and " social relations" played secondary roles. When the WHO 6-domain model was considered, we were surprised that, for such a conservative and religious culture, the " spiritual", psychological and social relations domains also did not have a direct impact on QOL. On the other hand, the direct predictors of QOL in our 5-domain model were "life satisfaction" and " sense of enjoyment", with social relations, environment, and physical/mental health playing secondary roles. These are not necessarily conflicting views, in the sense that the one model's views compliments that of the other. Hence, the WHOQOL-Bref can be used pragmatically from the perspective of the domains that emerge from the local culture, in comparison with the profile of WHO's four factors. This is in line with the understanding in SEM, that many models can exist in one data set. For instance, the WHO domain's emphasis on material circumstances is an indication that, in the poor economic circumstances of the people (as shown by the country's low GDP), the fulfilment of material needs (physical and environment domains) is a highly important contributor to the people's QOL. On the other hand, our 5-domain model's emphasis on "life satisfaction" (which includes the item, "life meaningful" that defines the "spiritual" domain of WHO) and "sense of enjoyment", recognizes the cultural emphasis on basic needs and spiritual matters and the outwardly suppressed individual innate urge for openness.

## Conclusion

In other words, the different valid models that exist in the WHOQOL-Bref can help us to understand the QOL characteristics of particular cultures or groups. In this instance, while the WHO model helps to define the group's main concerns of QOL from the comparative global perspective, the locally generated model gives us the more intimate local situation [4].

The findings add robustness to the credentials of WHO's 4-domain model as a universal QOL construct; while supporting the impression that analysis of WHOQOL-Bref could benefit from including all the items in factor analysis and using overall QOL as a dependent variable [5,11]. The clinical significance of these findings is that by more of such studies, a combination of domains from the WHO model and the local model can be generated and used to articulate more rigorous definitions of QOL, from which primary targets for subjective QOL interventions could be delineated that would have cross-cultural relevance.

## Competing interests

The author(s) declare that they have no competing interests.

## Authors' contributions

AWA and JUO jointly designed the study, analyzed the data and wrote up the manuscript. AMA and AJ played invaluable roles in data analysis and interpretation of data. All authors read and approved the manuscript.

## Pre-publication history

The pre-publication history for this paper can be accessed here:


